# Neglected Diseases and Poverty in “The Other America”: The Greatest Health Disparity in the United States?

**DOI:** 10.1371/journal.pntd.0000149

**Published:** 2007-12-26

**Authors:** Peter J. Hotez

**Affiliations:** Sabin Vaccine Institute and Department of Microbiology, Immunology, and Tropical Medicine, George Washington University Medical Center, Washington, D. C., United States of America

*To be sure, the other America is not impoverished in the same sense as those poor nations where millions cling to hunger as a defense against starvation. This country has escaped such extremes. That does not change the fact that tens of millions of Americans are, at this very moment, maimed in body and spirit, existing at levels beneath those necessary for human decency…They are without adequate housing and education and medical care.*

Michael Harrington

The Other America: Poverty in the United States, 1962

Michael Harrington's *The Other America: Poverty in the United States* was first published almost fifty years ago. His landmark book exposed to a wide audience the previously unseen poverty then rampant in many poor rural areas of the United States and in some of America's inner cities. Harrington was a widely read political writer and democratic socialist, and his manifesto on poverty was instrumental in stimulating President Lyndon B. Johnson's War on Poverty legislation and his Great Society programs.

In 1962, an estimated 40 million Americans lived in poverty, almost one-quarter of the US population. Today, the poverty rate in the US is roughly half of what it was when *The Other America* was first published, however, the total number of people living in poverty remains about the same. We now recognize that this group of 36.5 million impoverished Americans is at higher risk for heart disease, cancer, and other chronic diseases compared to the rest of the US population. However, it is not well known that just as the poorest people in the low-income countries of Africa, Asia, and Central and South America have the highest rates of the neglected tropical diseases (NTDs), there is evidence to suggest that large numbers of the poorest Americans living in the US also suffer from some of these unique infections.

During the early 20th century hookworm was a highly endemic soil-transmitted helminth infection in the American South, and a major cause of severe anemia and malnutrition in the region [1]. Together with malaria, niacin deficiency (pellagra), typhoid fever, ascariasis, trichuriasis, and other conditions common to areas of tropical and subtropical poverty, hookworm helped to foster the concept of the “sick man of the South” or the “lazy Southerner” [2]. The poverty-promoting aspects of these diseases are powerfully illustrated by the recent work of the economist Hoyt Bleakley, who has estimated that because of its impact on child growth and development, school performance, and school attendance, chronic hookworm infection in the American South was responsible for a 43% reduction in future wage-earning [3]. Hookworm is still one of the most important parasitic infections of humans in developing countries, although it is no longer a serious public health problem in the US [1]. A dramatic decline in the prevalence of NTDs in the American South, including hookworm, began in the 1930s when several New Deal programs transformed the region from an economy based on subsistence agriculture into an urbanized one with higher wage earning and improved quality dwellings [2].

Although it is widely believed that by now hookworm has been eliminated from the American South, we do not know this for sure. No large studies have been conducted since the 1970s, when it was shown that pockets of the infection still occurred in areas previously shown to be highly endemic [4]–[6]. Moreover, studies conducted during the late 1970s and 1980s revealed that significant numbers of American schoolchildren living in poor areas of the American South were infected with the large common roundworm, *Ascaris lumbricoides* [7]–[10], including 32% of school-aged children living in an unincorporated area of northern Florida [9], and an even higher percentage of poor Eastern Cherokee schoolchildren [10]. Similarly, when last studied, approximately 1%–4% of people living in rural Appalachia were determined to be infected with strongyloidiasis [11]. There is a high probability that ascariasis, trichuriasis, and strongyloidiasis are still important parasitic infections occurring in the US, but because they only occur among impoverished people and mostly underrepresented minorities, I believe that there has been a lack of political will to study the problem, so that these diseases of poverty have been allowed to simply remain neglected.

There are three other important NTDs for which we have more recent and robust data to document their impact on the health of the poorest Americans: toxocariasis, cysticercosis, and toxoplasmosis.

## Toxocariasis

Toxocariasis is a soil-transmitted helminth infection that can result in visceral larva migrans, visual impairment from ocular larval migrans, or a condition that resembles asthma, known as covert toxocariasis [12]. Urban playgrounds in the US have recently been shown to be a particularly rich source of *Toxocara* eggs [13], and inner-city children are at high risk of acquiring the infection. In inner-city areas of Bridgeport and New Haven, Connecticut, for example, the overall seroprevalence when measured during the 1990s was found to be 10% [14], but during the 1970s up to 30% of socioeconomically disadvantaged African Americans showed evidence of infection [15]. Based on both the published [12]–[15] and non-published literature (including a recent presentation by the Division of Parasitic Diseases of the US Centers for Disease Control and Prevention [CDC] at the 56th Annual Meeting of the American Society of Tropical Medicine and Hygiene held in November 2007, showing that the national seroprevalence of toxocariasis among the poor is 23%), I believe it is likely that hundreds of thousands of inner-city children (most of them African American and Hispanic children) are exposed regularly to this parasite. Because of its possible links to asthma, it would be important to determine whether covert toxocariasis is a basis for the rise of asthma among inner-city children in the northeastern US [14].

## Cysticercosis

Cysticercosis is another very serious parasitic worm infection and NTD, caused by the tapeworm *Taenia solium,* that results in seizures and other neurological manifestations. It is estimated that 1,000 to 2,000 new cases of neurocysticercosis are diagnosed annually in the US [16],[17]. At a reported incidence rate of eight to ten per 100,000 per year among Hispanic populations [17], as well as more recent data from a Hispanic community in Ventura County, California showing that the seroprevalence among adult Latinos is 2.8% [18], and considering that there are approximately 35 million Hispanics living in the United States, the number of actual cases may be much greater, possibly in the tens of thousands. In the hospitals of Los Angeles, California, neurocysticercosis currently accounts for 10% of all seizures presenting to some emergency departments [19]. Because cysticercosis is emerging as the leading cause of epilepsy among Hispanic populations, there is an urgent and important need for active surveillance studies of this infection.

## Toxoplasmosis

Toxoplasmosis is an important parasitic infection among Mexican Americans and African Americans [20]. If a pregnant mother becomes infected with *Toxoplasma* during her pregnancy, the newborn infant is at risk for congenital toxoplasmosis, a syndrome that can include mental retardation as well as vision and hearing loss. The US CDC estimates that approximately 400 to 4,000 infants are born with congenital toxoplasmosis annually in the United States [21]. Every one of these toxoplasmosis-infected infants represents a tragedy that could have been prevented, given the existence of studies showing that early diagnosis and treatment with antiparasitic drugs could improve outcome [22] and the availability of a newborn screening test, which is similar to the type used for the screening of phenylketonuria and other genetic disorders [23]. Currently only the states of Massachusetts and New Hampshire conduct newborn screening for toxoplasmosis.

We need to begin erasing these horrific health disparities by stepping up measures to conduct active and national-scale surveillance for soil-transmitted helminth infections, especially toxocariasis, as well as cysticercosis and congenital toxoplasmosis. In addition, based on data suggesting that the NTDs cutaneous leishmaniasis [24], ratborne leptospirosis and hantavirus infection [25],[26], dengue fever [27], brucellosis [28], tuberculosis caused by *Mycobacterium bovis* [29], trichomoniasis [30], and louse-borne trench fever [31] are emerging among the poor in the US, it is imperative that we address these conditions as well. In this issue of *PLoS Neglected Tropical Diseases* we describe a large number of imported cases of lymphatic filariasis, particularly among immigrants from sub-Saharan Africa, Asia, and tropical regions of the Americas [32].

The fact that reliable numbers on the actual prevalence of the NTDs are simply not available is reflective of their neglected status, and their disproportionate impact on minorities and poor people. There is an urgent need to support studies that (1) assess the disease burden resulting from the NTDs in the United States and (2) identify the minority populations at greatest risk, and then to (3) identify simple and cost-effective public health solutions. Accordingly, *PLoS Neglected Tropical Diseases* is pleased to consider and review articles on this vitally important topic. There are no excuses for allowing such glaring health disparities to persist in one of the world's wealthiest countries.
